# Repertory of the Back

**Published:** 1899-08

**Authors:** E. H. Wilsey

**Affiliations:** Parkersburg, W. Va.


### REPERTORY OF THE BACK.

E. H. Wilsey, M. D., Parkersburg, W. Va.

Continued from July No., p. 330.

### SACRUM.

**Pain** in sacral region, worse by rising from seat, *Lyco.*
— violent pains in sacrum, Carbo-an.
— pressive pains in sacrum, Carbo-an.
— in sacrum extending into feet, Kobalt.
— in sacral region, worse by sitting or bending backward, *Puls.*
— in right side of sacrum where it unites with the pelvis, Ver-v.
— as from a fracture in sacrum, Nat-m.
— in back as from a hot iron thrust through lower vertebræ, Alu.
— in sacrum passing into right thigh, Tellur.
— in sacrum from heavy lifting, Calc-c., *Sang-c.*
— in sacrum while walking, Borax.
— on right side of sacrum, Mez.
— in sacrum as if stiff, *Nit-ac.*
— commencing in sacral region and apparently going to uterus, Syph.
— in left side of sacrum, Sepia.
— in region of sacrum and loins, *Cimex-lec.*
**Paralysis**, a feeling of paralysis in sacrum, *Kalmia.*
**Paralytic** sensation in evening, Mag-m.
— pain in sacrum worse when straightening himself, Nat-m.
— and weak feeling at junction of lumbo-sacral region, *Phos.*
— bruised sensation in sacrum and knee joints, rising from seat, Verat.
**Piercing** in middle of sacrum, *Nat-s.*
— pain and tension in sacrum, Sul.
**Pimples**, on the sacrum and nates, Calc-c.

### SACRUM.

**Pinching** pains in sacrum with hysterical spasms, Caustic.
— frequent in the whole epigastrium, in the sides of the abdomen, and toward the sacrum, Nat-m.
— then shooting pains in the abdomen and sacrum especially morning and evening, Nitrum.
— about the navel and forcing down toward sacrum, then sudden urging to stool, soft yellow with a piece of tape-worm, Mag-m.
— and squeezing in sacrum all day, Nitrum.
— and cutting in stomach, toward the sacrum and left side, Nat-c.
**Plaster,** biting, burning, and sticking as from a mustard plaster between skin and flesh of sacrum in afternoon, Aloe.
**Plug,** severe pain in sacrum; she cannot sit, for then it seems as if there were a plug in her back; she must put a pillow under her, Carbo-v.
**Pressive** pain in sacrum when sitting, Caustic.
— pains in sacrum, Nit-ac., Carbo-an., Lyco.
— pain in sacrum while at rest, Euphorb.
— pain in sacrum as from fatigue in evening, *Puls.*
— bruised and sharp pressive pain in sacrum and in lumbar vertebræ, but especially in the junction of sacrum with pelvic bones, Iod.
**Pressive** pain in sacrum when standing and sitting as if from much stooping, Mur-ac.
— violently drawing pressive pain on left side near sacrum, Mez.
— tensive sensation in sacrum; deep internal when severe, with feeling in bone as if pressed asunder; worse when sitting and lying, *Berb.*
— burning in sacrum, somewhat to right, Stann.
**Pressed,** sensation in morning as if sacrum were being pressed inward from both sides, Kali-c.
**Pressing,** dragging pain over sacrum and at same time over hips; burning pressure in sacrum extending across dorsal

SACRUM.

region and under scapula like that produced by sewing, *Sepia.*

**Pressing** pain in sacrum, right side; later moves to middle of back, Lyssin.

— in sacral region only while walking, and particularly while putting the left foot down, Spong.

**Pressure**, cannot bear even pressure of soft pillow on sacrum, *Lob-i.*

— aching in sacrum in evening, better by pressure, *Sepia.*

— sharp pressure on and below sacrum, Sepia.

— pain in sacrum in evening; better by pressure on awaking from a nap; worse on left side in lumbar region all night, across loins when moving, Sepia.

— fullness in head and pressure in sacrum while sitting, with drowsiness at same time, Borax.

— oft repeated sharp pressure on sacrum and a little below it, Sepia.

— pain as from soreness in sacrum, with subsequent pressure in hypogastrium as if everything was coming out at the rectum and pudendum, Caustic.

— drawing pain in sacrum when standing, better by pressure, Ledum.

— pain in sacrum worse by standing, with pressure downward in hypogastrium, Lil-tig.

— in lumbo-sacral region and on rectum, Lil-tig.

— as with a dull point here and there internally as if near a bone; worse in evening on left side of sacrum, Lith-carb.

— bruised cramped pain in sacrum on pressure; sacral pain, *Podo.*

— drawing pressure in sacrum, extending to muscles on inside of ilium when standing, Sambucus.

— pain in sacrum so violent that it drew chest together, with pressure on stomach and constriction of abdomen, Lyco.

— dull shooting tearing in sacral region removed by pressure, Mag-m.

SACRUM.

**Pressure** in sacrum when standing in a stooping posture, Sul.
— in sacrum, Graph.
— tearing in sacrum, Carbo-veg.
— in abdomen on sacrum as from flatus, which passes off sparingly, affording some relief, Phos.
— pain in sacral region as from pressure of dull instruments, Convall.
— sacral sores, vitality of skin destroyed by pressure, *Peonia.*
— during menses intense pressure in the hypogastrium as if everything were coming out of the genital parts, with pain in sacrum; it drew downward in hips into lower limbs, Nit-ac.
— pressure in sacrum as of a heavy load as if it would burst, Agari.
— pressure in sacrum when standing, Tarax.
— outward pressure in left sacrum, Thuja.
— tension and weakness in lumbar and sacral region, Zinc.
**Pricking** and itching in sacrum when walking, *Merc.*
**Prolapsus** uteri, with dragging pains in sacrum, *Helon.*
**Prostration**, a feeling of prostration in sacrum at night, *Lith-c.*
**Pulsation** in sacrum, Nit-ac.
**Pulsating** stitch in os coccyx when sitting, and with stitches between scapulæ, Paris-q.
**Pustules** on sacrum, very sensitive when touched, Nat-c.
**Rectum**, dragging from sacrum toward rectum, Calc.
**Rest**, pain in sacrum only when bending backward, not while at rest, Kali-c.
— pain so severe as to prevent sleep or rest; at same time diphtheritic sore throat on right side, with sensation of a lump, could not swallow solid food, Lac-can.
— pressive pain in sacrum while at rest, Euphorb.
— pain in sacrum; more while walking than when at rest, Mez.
— sore pain in sacrum even when at rest, also when not touched, Nat-c.

SACRUM.

**Resting**, stiffness and lameness in sacrum; worse on resting after exercise, *Rhus.*

**Restlessness**, pain in sacrum as if broken, anxiety, restlessness, rush of blood most to the head, *Ars.*

**Riding**, carriage riding causes pain in sacrum, *Nux-mosch.*

**Rise**, pain in sacrum so that he could not rise again after sitting down, Calc-c.

— twitching pain in sacrum when stooping so he could not rise for a time, Kali-c.

— tensive pain in sacrum; worse in evening, so that he could not rise from his seat nor bend backward, *Bar-c.*

— painful stitches in sacrum; he can rise from a seat only with difficulty, Sul.

**Rising**, severe bruised pain in sacrum, chiefly in morning on rising, Kali-c.

— severe pain sacrum in morning on rising; going off on motion, Graph.

— pain in sacrum; worse from rising from a seat, *Lyco.*

**Rheumatic** pains in sacrum, *Oleum.*

**Rumbling** about abdomen, with forcing against the sacrum, Mag-m.

**Scratching**, itching worse by scratching on outer side of thigh, on sacrum and hips, succeeded by burning, Mag-m.

**Scrobiculum**, drawing from sacrum to right side of scrobiculum, *Chel.*

**Shooting**, acute squeezing pain in the left thigh, in morning on awaking, on turning over it extends to sacrum and terminates with shooting pains in heels, Nitrum.

**Severe** though brief pain in sacrum when rising from a seat, Petrol.

— sacral pains with labor-like pains in abdomen and discharge from vagina, Kali-c.

— constant drawing in sacrum and throbbing, better by lying down, Kali-c.

— pain in sacrum morning on rising, going off on motion, Graph.

SACRUM.

**Severe** pain in sacrum like a cutting through during movement and at rest, so that he could neither stand, walk, or lie down, Hepar.

— pain in sacral region extending to pubes, *Lauroc.*

— during menses severe pain in sacrum in morning on rising from bed so she could not move, Lyco.

— pain in sacrum, he cannot straighten up while sitting, he must bend forward, Lyco.

— pain and bruised sensation in sacrum and extremities, copious sweat with no relief, Eupat-perf.

— pain in sacrum so she could neither sit nor lie down, Calc-c.

**Sharp** pain in post-sacral region, Convall.

— sacral pains, Calc-phos.

— drawing transversely through the sacrum, very sensitive at every step, Carbo-an.

— oft-repeated sharp pressure on sacrum and a little below, Sepia.

— pressure and bruised pain sacrum and lumbar vertebræ, but especially at the junction of the pelvic bones it darts down into the lower limbs and causes a sort of limping in walking; the pain continues while sitting, standing, and lying, Hepar.

— tearing and sharp shooting in sacrum on moving, Dig.

**Shooting**, digging, shooting pain on left side sacrum, Dulc.

— jerking pain in sacrum, Euphor.

— pain in sacral bone at every expiration, Sul.

— frequent in sacrum when straightening himself up after stooping, Mur-ac.

— and pain in sacrum only when sitting and not when walking, Nat-c.

— in sacrum when he coughs, **Nit-ac.**

— Throbbing in sacrum at 6 P. M., with shooting, Sumbul.

— pinching, then shooting pains in abdomen and sacrum especially in morning and evening, **Nitrum.**

— pains shooting from sacrum down both hips, *Phyto.*

SACRUM.

**Shooting**, dull shooting, tearing in sacral region, removed by pressure, Mag-m.

— in sacrum extending down outside of legs to feet, Phyto.

— and tearing in sacral bones, Zinc.

— pain in sacrum so that he must keep perfectly quiet, worse from any motion, Coloc.

**Sitting**, tension and sensation of weakness in sacrum when sitting, with tension in head, Zinc.

— pain in sacral region, worse by sitting or bending backward, *Puls.*

— pain in sacrum like a great heaviness, rising suddenly when sitting and ceasing when moving about, Nat-c.

— pain in sacrum in sitting as if the catamenia would appear, Carbo-a.

— burning in sacrum when sitting, Borax.

— pain in sacrum so that he could hardly rise again after sitting, *Calc-c.*

— severe pain in sacrum, he cannot straighten up while sitting, but must bend forward, Lyco.

— tearing in sacrum transversely while sitting up straight, Lyco.

— pain in sacrum as from fatigue when stooping and when leaning back while sitting, Hepar.

— frequent pain just above sacrum when sitting, Kali-c.

— could not sit on account of most violent pain in sacrum, *Apis.*

— menses fourteen days too early, with pains especially violent in sacrum, worse when sitting, easiest while walking, Mag-c.

— before menses, straining and cutting in sacrum, with pain as if contracted and bruised, particularly while sitting, less when walking, Mag-c.

— pain in sacrum in sitting, Asaf.

— sensation when sitting as if something were darting through back toward sacral vertebræ, Aspar.

### SACRUM.

**Sitting**, pressive, tensive or pressing sensation in sacrum, deep internal when severe with feeling in bone as if pressed asunder, worse when sitting and lying, *Berb.*

— sticking in sacrum when sitting, worse by inspiration and expiration, Spig.

— a fine, tearing pain in sacrum while sitting, only passing from right to left side and upward, Spong.

— pain in sacrum after sitting a long time, Phos.

— pain in lumbar region and sacrum, especially during exertion in daytime and while sitting, *Agari.*

— pressure outward in left sacrum when sitting, Thuja.

— stitches in sacrum, worse when sitting than when in motion, Bar-c.

— pain in sacrum when sitting and stooping as from pressure, Borax.

— pain above the sacrum while walking, not while sitting, Sul.

— sprained pain in sacrum in morning in bed, also when sitting, Petrol.

**Sore** or burning pain near sacro-iliac symphysis, Rumex.

**Soreness** and pain in sacrum, Sarrac.

— pain in sacrum like soreness on moving, or like a spasmodic drawing, Sul-ac.

— of thighs as if beaten, with aching in sacral bones, CALC-PHOS.

— in tuberosities of ischia in a lean person, Selen.

— and pain in back and sacral region, *Sepia.*

— in sacro-iliac symphysis as if separated, *Calc-phos.*

— pain in sacrum as from soreness, with subsequent pressure in hypogastrium as if everything was coming out at the rectum and pudenda, Caust.

**Sore,** pain in sacrum even when at rest, also when not touched, Nat-c.

— pain in abdomen with pressure toward genitals as in menses, with pain in sacrum, Kali-c.

— sacral sores, vitality of skin destroyed by pressure, *Peonia.*

### SACRUM.

**Spot,** violent stitch on small spot between left ilium and sacrum, with slightest motion, has to lie on same spot without moving, *Calc-ph.*

— burning pain in a spot just above sacrum, Phos-ac.

**Sprained** pain in sacrum on motion, *Puls.*

— pain in hip beside sacrum when moving, Petrol.

— pain in left sacral region and hip when walking, Sul.

— pain in sacrum above hip in evening in bed and in afternoon, Sepia.

**Spreading** pain in sacrum, spreading over all parts of the body, Mez.

**Squeezing,** acute squeezing pain in left thigh in morning on waking; on turning over it extends to the sacrum and terminates with shooting pains in the heels, Nitrum.

— pinching in sacrum all day, Nitrum.

**Stand,** severe pain in sacrum like a cutting through during movement and at rest, so he could neither stand, walk nor lie down, Hepar.

— severe cutting in sacrum at the least motion, extending into the calves and feet so that he cannot walk, stand or lie down, Zinc.

**Standing,** griping in sacrum, worse by standing, better by walking, Merc.

— drawing pain in sacrum and sensation as if it were broken in walking, standing and lying, Carbo-an.

— pain in sacrum after standing or walking for a time, Kali-c.

— pain in sacrum when raising himself from a stooping position, and while standing, less while walking, Phos.

— pain in sacrum when standing, Ver-a.

— stitches through sacrum with drawing in lumbar vertebræ when standing, Con.

— dull pain in region of right ilium and sacrum while standing, Spong.

— drawing pain in sacrum when standing, better by pressure, Led.

SACRUM.

**Standing,** pain in sacrum, worse by standing, with pressure downward in hypogastrium, Lil-tig.

— stitch in sacrum, pain when standing, with confusion of head, *Lith-c.*

— intermitting tearing in sacrum after rising from stooping, while standing still quietly draws and jerks, Phos-ac.

— pressive pain in sacrum when standing and sitting, as if from much stooping, Mur-ac.

— cutting pain in sacrum in morning after rising and in evening before going to sleep, only when moving and bending down, not when standing upright, Petrol.

— pressure in sacrum when standing in stooping posture, Sul.

**Step,** sharp drawing transversely through the sacrum very sensitive to every step, Carbo-an., Acon.

**Sticking** in sacrum, Ign.

— burrowing on left side near sacrum, Dulc.

— outward and downward in sacrum, Lith-c.

— in sacrum when sitting, worse by inspiration and expiration, Spig.

— in sacrum on breathing, *Merc.*

— in right side of sacrum near false spinous processes, Merc.

— drawing, while walking, in sacrum, Bry.

— in left sacrum while walking, Calc-c.

— in sacrum at 5 P. M., Phelland.

**Stiff,** all joints are stiff in morning on rising, especially shoulders, sacral, and hips, Staph.

— back and sacrum stiff, cannot be bent after some exertion in riding, walking, and stooping, he can only raise himself slowly and with much difficulty, Lyco.

— pain in sacrum as if stiff, *Nit-ac.*

**Stiffness,** in sacrum and in hips, cannot walk straight, *Rheum.*

— and lameness in sacrum, worse on resting after exercise, *Rhus.*

— painful stiffness in sacrum, he can only rise from his seat with difficulty, Sul.

— tensive pain and stiffness in sacrum, Carbo-v.

### SACRUM.

**Stiffness** in sacrum, Kali-c., Lyco.
— in the lumbo-sacral articulation, Caustic.
— in sacrum on bending or beginning motion extending to hip-joint and thigh as if sinews were too short, *Lach.*
— drawing in sacrum with stiffness of back, Ledum.
— in scapula, hip-joints, and sacrum, Nat-m.
**Stitch** in sacrum occasionally, Nat-m.
— a stitch passing down the thigh at every breath, Carbo-an.
— a severe stitch in sacrum, Carbo-a.
— a sudden violent stitch in sacrum while walking in open air, *Agar.*
— a burning, startling stitch in sacrum, Mur-ac.
— a sudden stitch in sacrum in evening so that he could not move for a few minutes, Nat-c.
— severe stitch in sacrum after crouching down or rising up, Phos-ac.
— a stitch into the right ilium toward the sacrum, Mag-m.
— an occasional stitch from sacrum through left side of abdomen toward chest, Kali-c.
— burning stitch in sacrum causing one to start, *Mur-ac.*
— violent stitch in a small spot between left ilium and sacrum with slightest motion, has to lie on same spot without moving, *Calc-ph.*
— during coughing a stitch in sacrum, Nit-ac.
— transversely through sacrum, Cup.
— a stitch in rectum from the sacrum, Lyco.
— pain when standing with confusion in head and stitch in sacrum, *Lith-c.*
**Stitches**, in sacrum, Lyco., Iod., Mag-c., Nit-ac.
— attacks of stitches in sacrum, that take away the breath, with pain in the head and nape, followed by frequently alternating chill and heat, with anxiety about the scrobiculus cordis until evening, Sul.
— through sacrum to loins, Aloe.
— in sacrum on stooping, Verat-alb.

SACRUM.

**Stitches**, in sacrum when standing with drawing through lumbar region, Con.

— twitching stitches in sacrum and at same time on the leg above the ankle, Calc-c.

— at night pains in sacrum and stitches in both hips and in left side of chest, Lyco.

— along down sacrum to anus, Asaf.

— constant pulling stitches in sacrum, Berb.

— in sacrum, worse when sitting than when in motion, Bar-c.

— transversely across sacrum, Sul.

— sharp stitches right across sacrum, close above hips, Nat-m.

— close above the sacrum when taking a deep breath, Carbo-a.

— in sacrum and back, *Bry.*

— a weakness in sacrum mornings, a pain as if he had not sufficient strength in sacrum, stitches during certain movements, especially when walking, Calad.

— in sacrum, with drawing through the lumbar vertebræ when standing, *Con.*

— single itching stitches in sacrum, Caustic.

**Stitching**, crawling sensation, with stitching sacral pains, *Graph.*

— piercing jerking in back and sacrum, arresting the breath, Caust.

**Stooping**, pain in sacrum when stooping, Dig.

— pain in sacrum when straightening himself as after long stooping, Nat-m.

— violent pain in sacrum when and after stooping, Sarsa.

— pain in sacrum when sitting and stooping as from pressure, Borax.

— pain in sacrum; soon extends around hips and down right leg, with pressure and feeling of fatigue as from long stooping, *Bap.*

— after menses violent pain in sacrum as if bruised during stooping and at other times during the afternoon and evening, Mag-c.

— dull pain in sacrum while stooping, Borax.

SACRUM.

**Stooping**, stitches in sacrum on stooping, Ver-a.
— intermittent tearing in sacrum after rising from stooping down, while standing still it quietly draws in jerks, Phos-ac.
— momentary pain in sacrum, which for some time makes stooping and straightening one's self impossible, Nat-c.
— twitching pain in sacrum when stooping so that he could not raise himself for a time, Kali-c.
— bruised pain in sacrum on stooping and rising, Sul.
— pain in sacrum as from fatigue, and when leaning back while sitting and when stooping, Hepar.
— bruised feeling in left sacrum on stooping and on rising, Ver-a.
— pressive pain in sacrum while standing and sitting as from too much stooping, Mur-ac.
— violent pain in sacrum as after long stooping, Graph.
— pain in sacrum as after long stooping, Dulc.
— pain in sacrum when raising himself from a stooping position and in standing, less while walking, Phos.
**Stomach**, pinching and cutting in stomach toward the sacrum and left side, Nat-c.
— pain in sacrum so violent that it drew chest together, with pressure on stomach and constriction of abdomen, Lyco.
**Stool, a** uterine hemorrhage which came with every stool, with cutting in abdomen, and pains in sacrum and loins cease, Iod.
— pains in sacrum, with much discharge of mucus with stool, Borax.
— pain across sacrum, with urgency to stool, Amm-benz.
— pain in sacrum, with hard stool as if it would break, Lyco.
**Straining**, before menses, straining, cutting, and pain in sacrum as if contracted and bruised, particularly while sitting, less when walking, Mag-c.
— constant in sacrum toward rectum, Calc-c.
**Strain**, violent pain in sacrum as from a strain caused by lifting, worse on motion, Caustic.
**Straighten**, he cannot straighten for pain in sacrum, *Kali-bichro.*

